# Prognostic value of neutrophil-to-lymphocyte ratio in melanoma: Evidence from a PRISMA-compliant meta-analysis

**DOI:** 10.1097/MD.0000000000011446

**Published:** 2018-07-27

**Authors:** Yingguo Ding, Shan Zhang, Jianjun Qiao

**Affiliations:** Department of Dermatology, First Affiliated Hospital, College of Medicine, Zhejiang University, Hangzhou, Zhejiang, China.

**Keywords:** melanoma, meta-analysis, neutrophil-to-lymphocyte ratio, prognosis, survival

## Abstract

**Background:**

A number of studies have investigated the prognostic impact of the neutrophil-to-lymphocyte ratio (NLR) in patients with melanoma but the results were controversial. Therefore, we conducted a meta-analysis to explore the prognostic value of NLR in melanoma.

**Methods:**

The databases of PubMed, Embase, and Web of Science were thoroughly searched. Associations between NLR and overall survival (OS) and progression-free survival (PFS) were investigated by pooling hazard ratio (HR) and 95% confidence interval (CI).

**Results:**

A total of 12 studies comprising 3207 patients were finally included in the meta-analysis. The results showed that a high NLR was associated with poor OS (HR = 2.23, 95% CI = 1.64–3.04, *P* < .001, random-effects model) and PFS (HR = 2.19, 95% CI = 1.78–2.69, *P* < .001, fixed-effects model). Subgroup analyses demonstrated that NLR was still associated with poor OS and PFS for patients in Western countries who were treated with ipilimumab. No significant publication bias was found in this meta-analysis.

**Conclusion:**

This meta-analysis demonstrated that a high NLR was predictive of poor OS and PFS in patients with melanoma.

## 1 Introduction

Melanoma is the most aggressive form of skin cancer and the incidence of melanoma is still increasing.^[1,2]^ It is estimated that there will be 87,110 new cases and 9730 deaths from melanoma in 2017 in the United States.^[3]^ Several factors have been revealed to be predictive of poor survival in patients with melanoma, such as age, sex, Breslow tumor thickness, ulceration, and mitotic rate.^[4–6]^ Most patients presenting at a localized stage are potentially curable. However, patients with advanced melanoma have a poor prognosis, with a 5-year survival rate of 10%.^[7]^ Therefore, novel and efficient prognostic markers are of great importance to clinicians.

Inflammatory responses are considered to play important roles in tumor initiation and development.^[8,9]^ In recent years, hematologic parameters of the systemic inflammatory response have been shown to be of prognostic value in various cancers.^[10,11]^ These hematologic indices include C-reactive protein,^[11,12]^ the platelet-to-lymphocyte ratio,^[13,14]^ and the neutrophil-to-lymphocyte ratio (NLR).^[15–17]^ The NLR, which is presented as the number of circulating neutrophils divided by lymphocyte counts, has gained much attention.^[10]^ This is because the NLR is derived from routine blood tests and is cost free. A variety of studies also investigated the prognostic value of the NLR in melanoma but the results were inconsistent.^[18–22]^ The conflicting data among studies may be due to different patient populations, different regions, and various treatment methods. We thus collected the available data and conducted a rigorous quantitative meta-analysis to shed light on the prognostic role of the NLR in melanoma.

## 2 Materials and methods

### 2.1 Literature search

This study was designed and performed in accordance with the Preferred Reporting Items for Systematic Reviews and Meta-Analyses (PRISMA) statement.^[23]^ The electronic databases of PubMed, Embase, and Web of Science were searched updated to May 2018. The following search terms were used: “neutrophil-to-lymphocyte ratio” or “neutrophil-lymphocyte ratio” or “NLR” “melanoma∗” (MeSH) or “malignant melanoma∗” (MeSH). Other resources were also manually checked for potentially eligible studies. An ethical approval was not necessary since meta-analysis was based on secondary data.

### 2.2 Inclusion and exclusion criteria

Inclusion criteria for eligible studies were as follows: patients with pathologically confirmed melanoma; the data of overall survival (OS) or progression-free survival (PFS) was reported in the text or sufficient data were provided to calculate the HR and 95% confidence interval (CI) using Tierney method^[24]^; a definite cut-off value of the NLR was provided; the patients were in any stages; and articles published as full-text in English. The exclusion criteria were as follows: meeting abstracts, reviews, case reports, or letters; duplicate studies; studies lacking necessary information; and animal studies. Two investigators (YD and SZ) independently evaluated all the candidate articles. Disagreements were resolved by discussion. OS was calculated from date of treatment initiation to the date of death from any cause of disease. Patients who were still alive were censored at the last follow-up. PFS was calculated from the date of treatment initiation until progression, as documented by imaging, according to response evaluation criteria in solid tumors or clinical examination or death.

### 2.3 Data extraction and quality assessment

The following information was extracted from each eligible study: name of the first author, year of publication, study country, sample size, study period, sex, mean/median age, study design, tumor stage, treatment methods, cut-off value of the NLR, HR, and 95% CI for OS and/or PFS. Quality assessment for the included studies was performed according to the Newcastle–Ottawa scale (NOS).^[25]^ The full score is 9 points and studies with ≥6 points were considered high-quality studies.

### 2.4 Statistical analysis

The pooled HR with its 95% CI was utilized to quantitatively assess the prognostic significance of the NLR for melanoma patients. Cochrane *Q* and *I*^2^ tests were used to evaluate the heterogeneity among studies. *P* < .10 for the *Q* test or *I*^2^ > 50% indicates significant heterogeneity and the random-effects model (DerSimonian–Laird method) is utilized in that situation. Otherwise, the fixed-effects model (Mantel–Haenszel method) is chosen. Subgroup analyses were conducted to examine the prognostic value of the NLR in different populations. Sensitivity analyses were performed to confirm the stability of the results. Begg funnel plot test^[26]^ was used to evaluate the publication bias. Stata 12.0 software (Stata Corp, College Station, TX) was used for all statistical analyses. *P* < .05 was considered statistically significant.

## 3 Results

### 3.1 Study selection and characteristics

**Figure 1:**
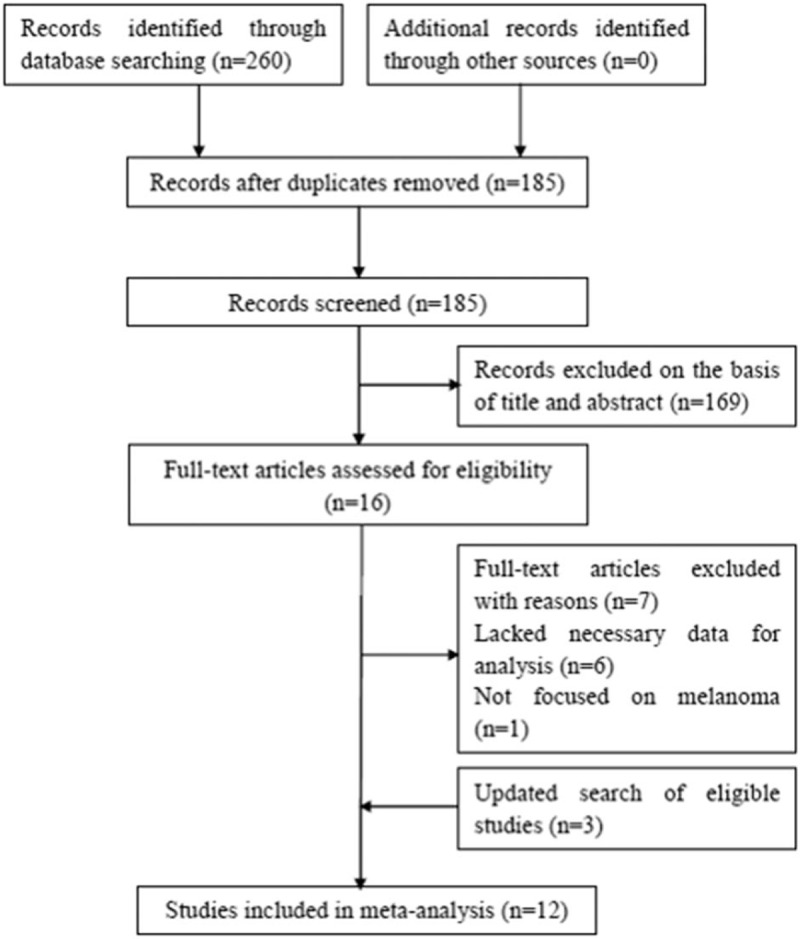
Flowchart of literature search.

The initial search retrieved 260 studies. As shown in Figure 1, after duplicate records were removed, 185 records were left. After screening the titles or abstracts, 169 studies were discarded because they were animal studies, reviews, meeting abstracts, or irrelevant studies. Next, 16 full-text articles were further evaluated. Seven studies were excluded because they lacked necessary data or did not focus on melanoma. We then updated the search process and 3 eligible studies were added on May 2018. Finally, 12 studies^[18–22,27–33]^ published between 2014 and 2018 were included in this meta-analysis. The characteristics of the included studies are shown in Table 1. The sample sizes ranged from 44 to 1431 and the total sample size was 3207. Four studies^[21,28,32,33]^ were conducted in the United States, 2 studies^[19,20]^ were performed in Italy, and 1 study was carried out in each of the United Kingdom,^[18]^ France,^[27]^ Korea,^[29]^ Mexico,^[30]^ France,^[31]^ and China.^[22]^ Five studies^[18–20,28,29]^ selected 5 as the cut-off value of the NLR and other studies used 4,^[27,31]^ 3,^[21]^ 2.5,^[32]^ 4.73,^[33]^ 2,^[30]^ and 2.35.^[22]^ The NOS scores of the studies ranged from 6 to 9, with a median value of 8.

**Table 1:**
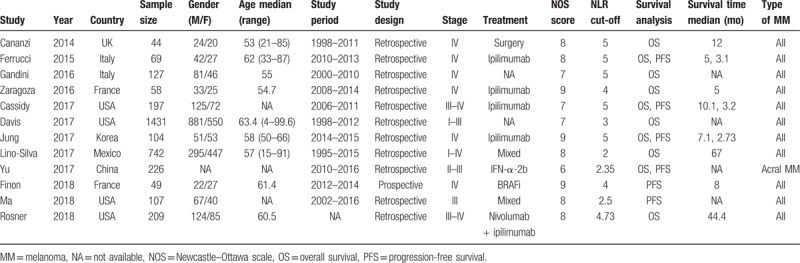
Characteristics of the included studies for meta-analysis.

### 3.2 Prognostic value of NLR for OS

**Figure 2:**
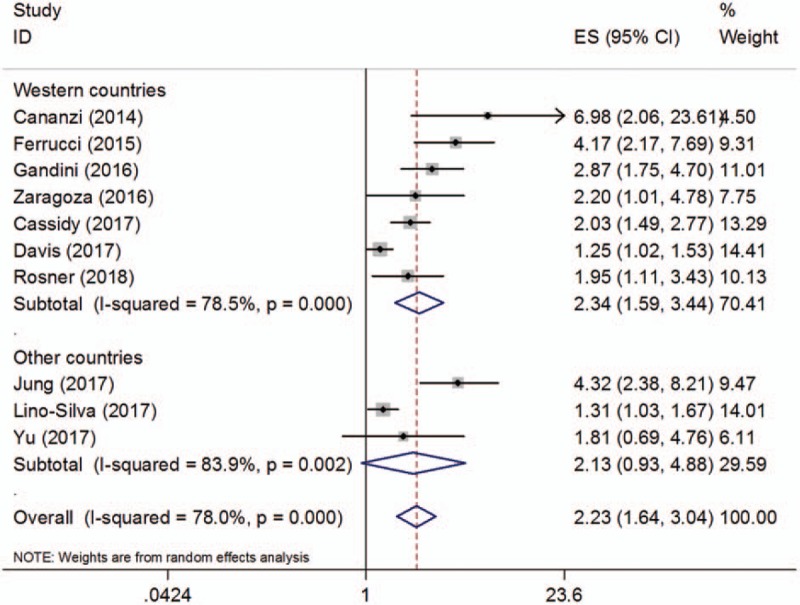
Pooled hazard ratio value for overall survival, subgroup analysis of study location.

Ten studies^[18–22,27–30,33]^ presented the hazard ratio (HR) and 95% CI for OS. As shown in Figure 2, the pooled results were HR = 2.23, 95% CI = 1.64 to 3.04, *P* < .001 (random-effects model), because the incidence of melanoma is quite different in Caucasian countries and non-Caucasian countries. Among non-Caucasian populations, incidence rates are quite variable and relatively low. Moreover, in this meta-analysis, most eligible studies were from Caucasian countries; therefore, we conducted subgroup analysis between Western countries and other countries.^[34]^ The subgroup analyses showed that NLR was still associated with poor OS for patients in Western countries (HR = 2.34, 95% CI = 1.59–3.44, *P* < .001, random-effects model; Fig. 2, Table 2). Furthermore, the prognostic role of NLR for OS remained consistent irrespective of the treatment method (ipilimumab vs other methods) or cut-off value (NLR = 5 vs ≠5) (Table 2).

**Table 2:**
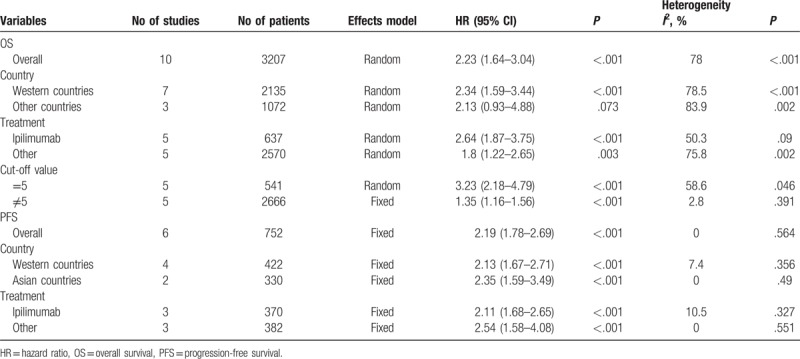
Main results of the meta-analysis.

### 3.3 Prognostic value of NLR for PFS

**Figure 3:**
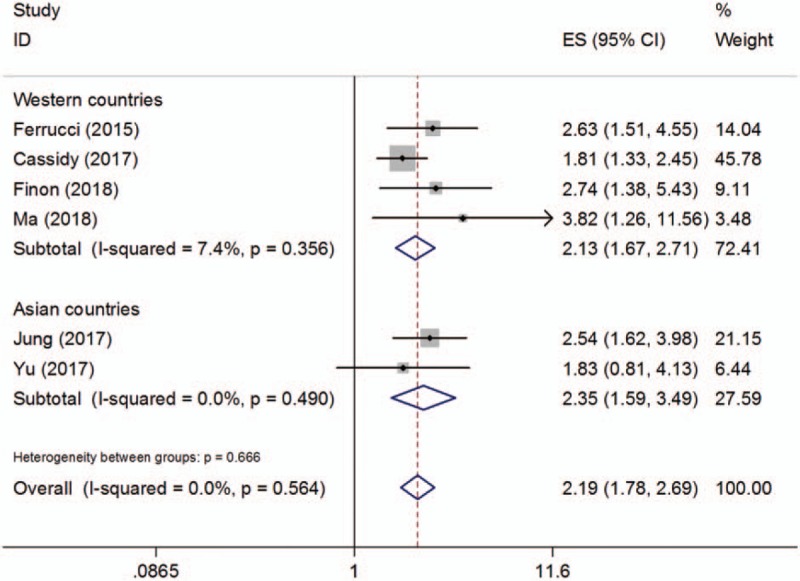
Pooled hazard ratio value for progression-free survival, subgroup analysis of study location.

Six studies^[19,22,28,29,31,32]^ with a total of 752 patients investigated the correlation between the NLR and PFS. The combined results are shown in Figure 3 and Table 2. The pooled HR and 95% CI were HR = 2.19, 95% CI = 1.78 to 2.69, *P* < .001 (fixed-effects model). The subgroup analyses demonstrated that an elevated NLR indicated a poor PFS in both Western countries (HR = 2.13, 95% CI = 1.67–2.71, *P* < .001) and Asian countries (HR = 2.35, 95% CI = 1.59–3.49, *P* < .001). In addition, a high NLR was associated with shorted PFS for patients treated with ipilimumab (HR = 2.11, 95% CI = 1.68–2.65, *P* < .001) and other treatment methods (HR = 2.54, 95% CI = 1.58–4.08, *P* < .001). Non-significant heterogeneity was detected for all analyses of the NLR and PFS.

### 3.4 Sensitivity analysis

**Figure 4:**
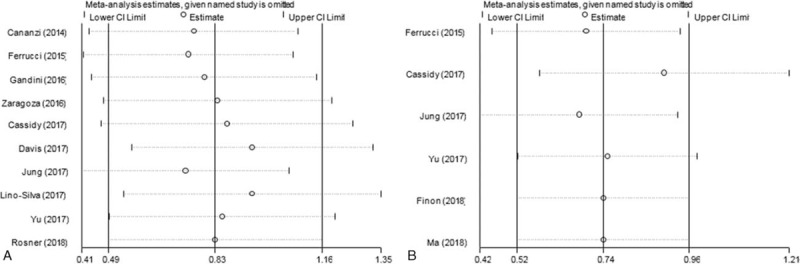
Sensitivity analyses for (A) overall survival and (B) progression-free survival.

Sensitivity analysis was conducted by excluding each included study by turn and then calculating the pooled results. As shown in Figure 4, the pooled results of both OS and PFS did not significantly change in sensitivity analysis, indicating the robustness of the results of this meta-analysis.

### 3.5 Publication bias

**Figure 5:**
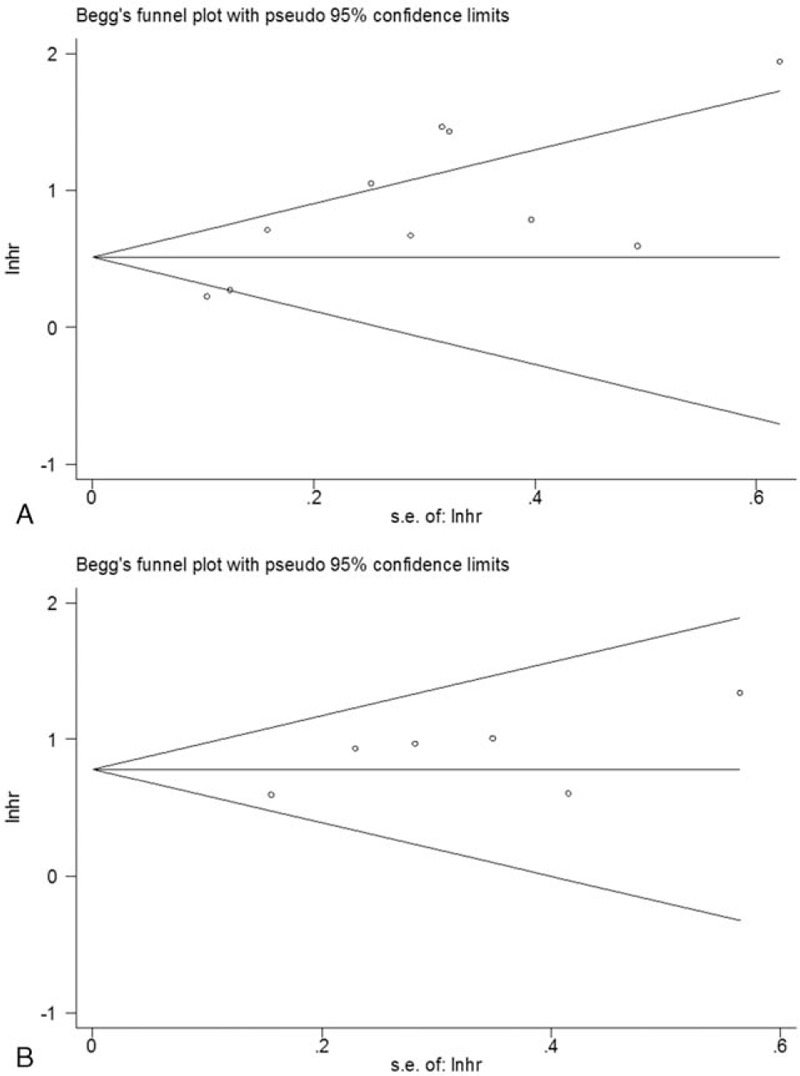
Begg funnel plots for (A) overall survival and (B) progression-free survival.

Publication bias was examined using Begg funnel plot. The *P*-values for Begg test were *P* = .21 for OS and *P* = .707 for PFS (Fig. 5). The results suggested that there was no statistically significant publication bias in this study.

## 4 Discussion

In this meta-analysis, we found that an elevated NLR was predictive of poor OS and PFS in patients with melanoma. Moreover, a high NLR was associated with poor OS and PFS in patients from Western countries and for patients treated with ipilimumab. NLR = 5 is the most commonly used cut-off value for melanoma. The results of sensitivity analysis and the publication bias test confirmed the reliability of this meta-analysis. This study demonstrated that the NLR could be an efficient prognostic marker for melanoma.

Accumulating evidence has demonstrated that inflammation was involved in the process of tumor progression.^[8,9]^ Recent studies have revealed that the NLR could reflect the balance between tumor-promoting inflammation and anti-tumor activity.^[10]^ Neutrophils and lymphocytes are important cell types that reflect systemic immune responses. Neutrophils play important roles in tumor progression.^[35–37]^ They are considered the primary source of vascular endothelial growth factor, which promotes tumor angiogenesis.^[38]^ Furthermore, tumor-associated neutrophils can contribute to tumor metastasis by enhancing the seeding of tumor cells.^[37]^ In contrast, lymphocytes are immune cells and exhibit antitumor activity.^[39]^ Lymphocytes could induce cytotoxic cell death and suppress tumor cell proliferation and progression.^[40]^ Previous studies have shown that lymphocytes are barriers to tumor migration.^[15]^ Therefore, the NLR, which combines the neutrophil count and lymphocyte count, is biologically reasonable and is predictive of poor survival outcomes for a variety of cancers.^[41–45]^

The present meta-analysis showed that a high NLR was an unfavorable factor for both OS and PFS in melanoma. A number of previous meta-analyses also explored the prognostic value of NLR in various solid tumors.^[41,43,44,46,47]^ For example, Gu et al indicated that an elevated pretreatment NLR might be a predictive factor for a poor prognosis in non-small-cell lung cancer patients.^[48]^ Wei et al demonstrated that breast cancer patients with a higher NLR had poorer prognoses.^[46]^ Wang et al also showed that a high NLR has a strong association with worse OS and PFS in patients with diffuse large B-cell lymphoma.^[45]^ A comprehensive meta-analysis of 100 studies showed that a high NLR is associated with an adverse OS in many solid tumors.^[10]^ However, in that study, melanoma was not included and the prognostic value of the NLR in melanoma was not investigated in this meta-analysis. To the best of our knowledge, this study is the first meta-analysis investigating the pooled results of the prognostic role of the NLR for patients with melanoma. We noticed a recent study^[49]^ similar to our work. We carefully read the paper and found that our study was different with Sacdalan's study in the following aspects. First, Sacdalan's study is a review and meta-analysis; however, our manuscript is a meta-analysis in accordance with PRISMA guideline. Second, Sacdalan's study only included patients receiving immune checkpoint inhibitors, while our study did not limit the treatment methods. Third, our manuscript only included full-text studies, while Sacdalan's study included several meeting abstracts. Taken together, compared with Sacdalan's study, our study is conducted with strict guideline with recruitment of more comprehensive patient population. In addition, of all eligible studies, only Yu's work recruited acral melanoma patients, while the other studies recruited all types of melanoma. Therefore, the results of this study are applicable to all types of melanoma. We also noticed that the patients were on early and advanced stages. Because most of the eligible studies included patients in advanced stages, the current meta-analysis may be more suggestive to advanced patients.

Several limitations still need to be noted in our study. First, significant heterogeneity was detected in the analysis between the NLR and OS. Although we adopted a random-effects model for analysis, heterogeneity is a universal problem in meta-analysis. Second, most eligible studies are conducted in Western countries. Therefore, the results may be more applicable for Caucasian patients and more studies on other ethnic backgrounds are still required. Third, the cut-off values of the NLR were inconsistent in the included studies, which might cause selection bias. Fourth, only 12 studies were included. The sample is small and the subgroup analysis of PFS sometimes only contained 3 studies. Further studies on the NLR and melanoma are still required.

In summary, this meta-analysis demonstrated that a high NLR was predictive of poor OS and PFS in patients with melanoma. For patients in Western countries and those who are treated with ipilimumab, the NLR has consistent prognostic significance. Because of the above-mentioned limitations, future studies should recruit patients with diverse ethnicities and use a uniform cut-off value to validate the results of our meta-analysis.

## Author contributions

**Conceptualization:** Yingguo Ding.

**Data curation:** Yingguo Ding.

**Formal analysis:** Yingguo Ding, Shan Zhang, Jianjun Qiao.

**Funding acquisition:** Yingguo Ding, Shan Zhang.

**Investigation:** Yingguo Ding, Shan Zhang.

**Methodology:** Yingguo Ding, Jianjun Qiao.

**Project administration:** Yingguo Ding.

**Resources:** Yingguo Ding, Shan Zhang, Jianjun Qiao.

**Software:** Yingguo Ding, Jianjun Qiao.

**Supervision:** Yingguo Ding, Jianjun Qiao.

**Validation:** Yingguo Ding.

**Visualization:** Yingguo Ding.

**Writing – original draft:** Yingguo Ding, Shan Zhang.

**Writing – review & editing:** Yingguo Ding, Jianjun Qiao.
